# Supplementary material for: Super-resolution microscopy reveals coupling between mammalian centriole subdistal appendages and distal appendages
Source: eLife. 2020 Apr 3;9:e53580. doi: 10.7554/eLife.53580 (PMC7173962; doi:10.7554/eLife.53580)
Supplement: Supplementary file 1. — Radial and longitudinal positions of DAP and sDAP proteins. The positions of DAP and sDAP proteins from dSTORM images (relative to the DAP protein SCLT1) in the present study and our previous work (Yang et al., 2018). Supplementary table 2. Primary antibodies used in immunofluorescent staining. Summarized information on the sources, immunogens, and conditions of the primary antibodies used in the present study. [file elife-53580-supp1.docx]

**SUPPLEMENTARY FILE 1**

**Supplementary Table 1** Radial and longitudinal position of DAP and sDAP proteins

| Protein | Diameter  (mean ± SD, nm) | | Longitudinal position relative to SCLT1 (mean ± SD, nm) | |
| --- | --- | --- | --- | --- |
| FBF1 | 429 ± 23 | (12 MCs) | 30 ± 11 | (10 MCs) |
| CEP164 | 381 ± 70; 475 ± 39 | (15 MCs) | -35 ± 7; 26 ± 9 | (6 MCs) |
| SCLT1 | 419 ± 36 | (8 MCs) | 0 ± 8 | (6 MCs) |
| CEP89 | 352 ± 45 | (8 MCs) | -106 ± 28;  27 ± 7 | (12 MCs) |
| CEP83 | 315 ± 20 | (13 MCs) | -59 ± 0 | - |
| ODF2 | 249 ± 52 | (8 MCs) | -95 ± 26;  -198 ± 40 | (8 MCs) |
| CEP128 | 305.8 ± 66 | (10 MCs) | -149 ± 41 | (10 MCs) |
| Centriolin | 360 ± 46 | (9 MCs) | -127 ± 64 | (7 MCs) |
| CEP170 | 527 ± 75 | (7 MCs) | -111 ± 46 | (7 MCs) |
| Ninein | 512 ± 57 | (9 MCs) | -122 ± 56 | (7 MCs) |
| CCDC68 | 371 ± 93 | (7 MCs) | - | - |

MC: mother centriole

**Supplementary table 2** List of primary antibodies used in immunofluorescent staining

| Name of Antibody | Host | Company | Cat # | Dilution | Immunogen (sequence) | Reference |
| --- | --- | --- | --- | --- | --- | --- |
| FBF1 | rabbit IgG | Proteintech, Rosemont, IL, USA | 11531-1-AP | 1/200 | hFBF1 (20-347 aa) | Wei, Xu et al. 2013 |
| SCLT1 | rat IgG | Tanos et al. *Genes & development* (2013) | - | 1/250 | - | Tanos, Yang et al. 2013 |
| CEP89 | rat IgG | Tanos et al. *Genes & development* (2013) | - | 1/500 | - | Tanos, Yang et al. 2013 |
| ODF2 or ODF2-N | rabbit IgG | Sigma-Aldrich | HPA001874 | 1/200 | hODF2 (39-200 aa) | Asante, Maccarthy-Morrogh et al. 2013 |
| ODF2-C | rabbit IgG | Abcam | ab43840 | 1/200 | hODF2 (800aa to C terminus) | Kashihara H et al, 2019 |
| CEP128 | rabbit IgG | Abcam | ab118797 | 1/200 | hCEP128 (1044-1094 aa) | Gregory Mazo et al. 2017 |
| CENTRIOLIN | mouse IgG | Santa Cruz | sc-365521 | 1/200 | hCentriolin (2026-2325 aa) | Gregory Mazo et al. 2017 |
| NINEIN | rabbit IgG | Bethyl | A301-504 | 1/1000 | hNIN (1850-1900 aa) | Gregory Mazo et al. 2017 |
| NINEIN | mouse IgG | Santa Cruz | sc-376420 | 1/500 | hNIN (289-476 aa) | Ou,Y.Y.,etal.2002 |
| CEP170 | rabbit IgG | Abcam | ab72505 | 1/400 | hCEP170 (1534-1584 aa) | Shumilov A et al. 2017 |
| CCDC68 | rabbit IgG | Proteintech | 26301-1-AP | 1/400 | CCDC68 (1-335 aa) | Huang et al. 2017 |
| C-NAP1 | mouse IgG | Santa Cruz | sc-390540 | 1/200 | hC-NAP1 (107-286 aa) | Hsu, W.H., et al. 2018 |
| g-tubulin | mouse IgG | Sigma-Aldrich | T6557 | 1/500 | N-terminal amino acids of γ-tubulin | Joshi HC, et al. 1992 |
| a-tubulin | mouse IgG | Santa Cruz | sc-32293 | 1/500 | native chick brain microtubules | Grundmann, M., et al. 2018. |
| Centrin | mouse IgG | Millipore | 04-1624 | 1/400 | C-terminus of Chlamydomonas Centrin | Kim, S, et. al, 2015 |
